# Diversity-Oriented Synthesis as a Tool for Chemical Genetics

**DOI:** 10.3390/molecules191016506

**Published:** 2014-10-14

**Authors:** Elena Lenci, Antonio Guarna, Andrea Trabocchi

**Affiliations:** Department of Chemistry “Ugo Schiff”, University of Florence, via della Lastruccia 13, I-50019 Sesto Fiorentino, Florence, Italy

**Keywords:** diversity-oriented synthesis, chemical genetics, drug discovery, chemical biology, small molecules, high-throughput screening

## Abstract

Chemical genetics is an approach for identifying small molecules with the ability to induce a biological phenotype or to interact with a particular gene product, and it is an emerging tool for lead generation in drug discovery. Accordingly, there is a need for efficient and versatile synthetic processes capable of generating complex and diverse molecular libraries, and Diversity-Oriented Synthesis (DOS) of small molecules is the concept of choice to give access to new chemotypes with high chemical diversity. In this review, the combination of chemical genetics and diversity-oriented synthesis to identify new chemotypes as hit compounds in chemical biology and drug discovery is reported, giving an overview of basic concepts and selected case studies.

## 1. Introduction

Drug discovery is a key field of pharmaceutical industries. After an impressive growth at the end of the last century, the number of new molecular entities launched on the market dramatically decreased in recent years, so that it has been claiming that the “ice age” of drug discovery is approaching [1]. Although target-based drug discovery approach remains the “gold standard” in hit identification, a fundamental challenge has emerged. Many disorders, such as cancer and neurodegenerative diseases, are often associated with complex interactions, such as those involving transcription factors, protein-protein interactions (PPIs) and DNA-protein interactions [2]. As a consequence of that, such targets have been termed “undruggable”, due to the difficulty in being applied to typical drug-screening programs [3].

**Figure 1:**
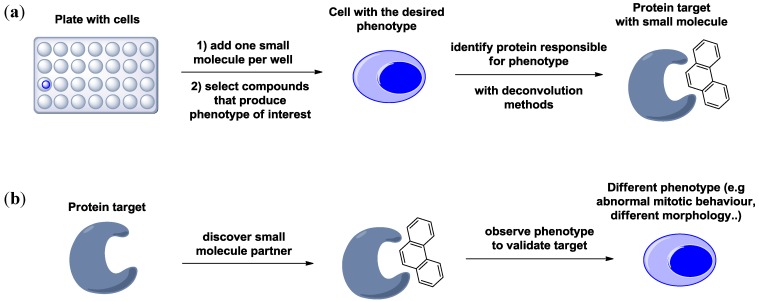
(**a**) Forward chemical genetics approach; (**b**) Reverse chemical genetics approach.

In this context, chemical genetics [4,5,6] is a brilliant example of a methodological development in lead generation. It uses small molecules to perturb the function of gene products, thus facilitating the dissection of biological processes. Similarly to genetics, chemical genetics can be divided into “forward” and “reverse” approach (Figure 1). In forward chemical genetics, a small molecule eliciting a desired phenotype is identified, and its protein partner is discovered subsequently following a deconvolution method. These studies (from molecule to protein to phenotype) are exploited when the aim of the investigation is the identification of molecules able to induce a specific biological effect. On the other hand, reverse chemical genetics approaches are fundamental to validate a known target. These studies involve functional small-molecule assays targeted directly to a protein of interest. Once a compound targeting a given protein is identified, the challenge is to discover if the small molecule has an effect in a cellular context. In fact, there is no guarantee that the compounds will affect a protein in a way that results in a functional phenotypic outcome in the cell. Indeed, targeting a specific protein may not result in giving the desired therapeutic consequence, and a considerable effort is paid to validate the target prior to executing full-scale screening. Nevertheless, forward chemical genetics is the most attractive and the most used approach in chemical biology, because it allows for the discovery of both new targets and new lead compounds with potential therapeutic applications [7,8,9].

### 1.1. Plasmodium Falciparum: An Example of the Application of Both Forward and Reverse Chemical Genetics Studies

**Figure 2:**
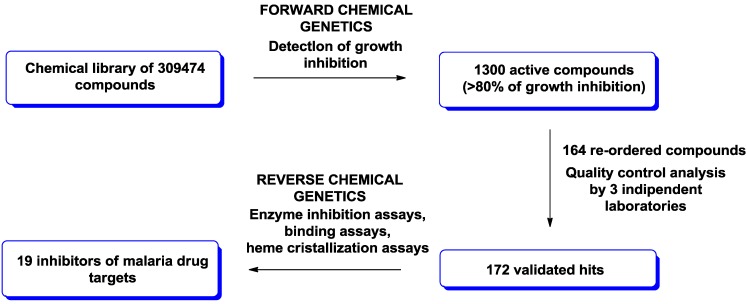
Illustration of forward and chemical genetics approaches used by Guy and co-workers for malaria drug discovery [14].

The application of chemical genetics in drug discovery programs was demonstrated being particularly powerful in the search for new antimalarials [10,11,12,13]. Guy and co-workers have done a significant contribution for new chemotypes discovery in this area, using both forward and reverse chemical genetics approaches (Figure 2) [14]. The authors screened a library of 309474 compounds (including drugs, enzyme inhibitors, natural products, *etc.*) against *Plasmodium falciparum* strains, according to a forward chemical genetics approach. This primary screen revealed 1130 compounds able to induce more than 80% of growth inhibition. From this set, 228 small molecules were re-purchased and re-tested by three independent laboratories, providing 172 cross-validated hits.

These active compounds were then subjected to a reverse chemical genetics study, using 66 potential antimalarial targets. In particular, the authors investigated the inhibition activity of these compounds against dihydroorotate dehydrogenase (Pf-DHOD) [15], falcipain-2 cystein protease (Pf-FP2) [16] and dihydrofolate reductase (Pf-DHFR). Additionally, they evaluated heme detoxification and hemozoin formation inhibition, using heme crystallization assay [17,18]. To expand the pool of potential targets, these 172 compounds were then studied for binding to 61 recombinant malarial proteins, using thermal-melt shift assays. In this way, 19 new inhibitors of malaria drug targets were discovered.

### 1.2. Generating Libraries for Chemical Genetics Studies. The Importance of Diversity-Oriented Synthesis

There is no technology enabling a chemical genetics approach to be applied generally. A chemical genetics study requires: (1) the design and synthesis of a chemical library; (2) the screening of the library in the system of choice; (3) target identification/validation. Thus, in the first step of the chemical-genetics process, it is necessary to assemble a library of different and potentially bioactive small molecules [19]. During the last decades, organic chemists have taken advantage of a significant number of high-throughput synthesis methods, such as solid-phase techniques [20,21] and combinatorial chemistry [22,23].

Unfortunately, despite the success in several drug discovery programs, the combinatorial chemistry approach has not fulfilled the desired expectations in difficult-to-target areas, such as those addressing protein-protein and DNA-protein interactions. Most of the combinatorial libraries are prepared through the functionalization of a common skeleton, so the compounds therein generated, often possessing flat structures and few stereocenters, showed limited structural complexity and diversity. The structural features common to diverse classes of bioactive natural products confirm that three-dimensional complexity is necessary for interacting with biological macromolecules. Furthermore, libraries that interrogate larger areas of chemical space increase the chance of identifying novel lead compounds [24].

In this context, Diversity-Oriented Synthesis (DOS) [25,26], which aims to synthesize the largest number of structurally complex small molecules, was conceived as a novel concept for the construction of libraries mainly addressing drug discovery issues. DOS has been defined by Spring as “the deliberate, simultaneous and efficient synthesis of more than one target compound in a diversity-driven approach” [27]. Since Schreiber’s seminal paper [28], several DOS strategies have been proposed in order to achieve three different types of molecular diversity, namely appendage, stereochemical and skeletal diversity [29,30]. Such strategies are usually divided in: (1) reagent-based approaches, where many distinct compounds are obtained subjecting a starting molecule to different reaction conditions; and (2) substrate-based approaches, where different starting materials, containing pre-encoded skeletal information, are transformed to distinct products using the same reaction conditions. Additionally, a very useful strategy, proposed by Schreiber and Nielsen, is the build/couple/pair approach [31]. Building blocks are assembled in polyfunctional intermediates, which can undergo different intramolecular cyclizations, thus achieving complex scaffolds in a divergent fashion. This approach exploits efficient and modular synthetic routes, typically composed by no more than five steps, so once interesting activities of some compounds are ascertained, focused libraries around such hit structures can be conveniently generated in a follow-up process.

**Figure 3:**
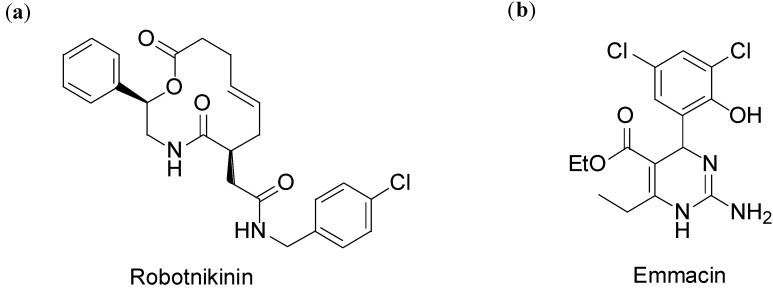
(**a**) Robotnikinin, a low µM sonic hedgehog inhibitor discovered by Schreiber’s group [33]; (**b**) Emmacin, a potent antibacterial compound with MIC_50_ on the low µg/mL range discovered by Spring’s group [34,35].

Nevertheless, the chemical genetics approach combined with DOS synthetic strategy is currently somewhat limited. The emergence of international screening initiatives, such as The Society for Laboratory Automation and Screening (SLAS), EU-OPENSCREEN or ChemBioNet, has increased significantly the number of high-throughput screening studies conducted on DOS libraries. Most drug discovery initiatives rely, however, on screening compound collections for their activity against known biological targets [32]. For example, Schreiber and co-workers discovered a potent sonic hedgehog inhibitor with a K_D_ value of 3.1 µM, robotnikinin (Figure 3a) from the screening of a DOS library of 2070 amino alcohol-derived macrocycles with the Small Molecule Microarray (SMM) technology [33]. The Spring’s group, instead, screened a DOS library of 223 compounds, based on 30 distinct molecular scaffolds, using high-throughput phenotypic assays, thus identifying emmacin (Figure 3b) as a potent antibacterial compound showing growth inhibitory activity against epidemic methicillin-resistant strains of *S. aureus* (EMRSA-15 and -16) with MIC_50_ values of 9 µg/mL [34,35].

On the contrary, there are only few examples of reverse and forward chemical genetics programs based on DOS libraries. In order to make new chemical genetics approaches time- and money-saving a double effort is required: the set-up of an easy, rapid and versatile chemical synthesis and the development of a rapid, efficient and economical screening process. These tools are used mostly for drug development, rather than on the dissection of biological systems, especially in pharmaceutical industries. However, these technologies have become sufficiently robust and wide-spread, that we can now conceive their use to study virtually any biological process. Dissecting biological systems with small molecule probes became increasingly popular, as it holds the promise of discovering new targets outside the “druggable” genome.

In the present work, we describe three selected case studies that illustrate the power of diversity-oriented synthesis as a tool for chemical genetics, focusing on the challenges of designing and manufacturing compound libraries.

## 2. Case Study 1: Pioneering Work of Stuart L. Schreiber

**Scheme 1:**
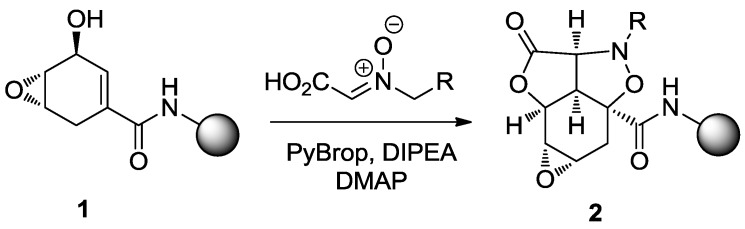
Synthesis of the tetracyclic scaffold **2** with tandem acylation/1,3-dipolar cycloaddition [36].

The pioneering work of Schreiber, published in 1999, still remains one of the most important examples of a striking DOS synthesis for chemical genetics and cell-based assays [36,37]. Starting from the simple shikimic acid derivative **1**, Schreiber and co-workers were able to generate the tetracyclic natural product-like scaffold **2**, using a tandem acylation/1,3-dipolar cycloaddition with an array of nitrone-carboxylic acids [38] (Scheme 1).

**Scheme 2:**
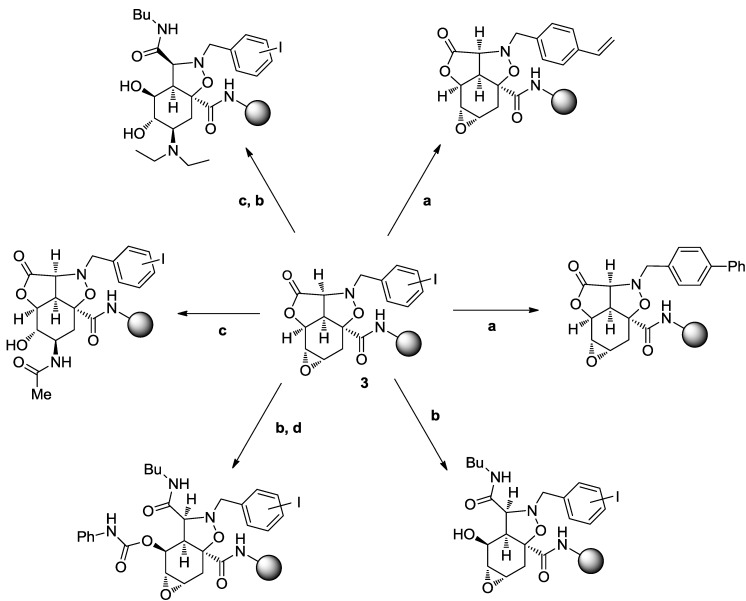
Potential sites of functionalization and representative examples of compounds derived from template **3** by (**a**) cross-coupling reactions, (**b**) aminolysis of lactone moiety, (**c**) epoxide ring opening and (**d**) alcohol esterification [36,37].

As shown in Scheme 2, the tetracyclic template **2** is a rigid and densely functionalized compound, which can be decorated with a variety of appendages. The introduction of benzyl iodide substituents on the isoxazoline nitrogen allows for the application of a wide number of different palladium-catalyzed reactions (e.g., cross coupling, amination, etherification, carbonylation), even though the authors demonstrated that only Suzuki [39], Stille [40] and Sonogashira/Castro-Stephens [41,42] reactions were successfully performed on template **3** (Scheme 2a). Additionally, the electrophilic lactone and epoxide moieties can react with nucleophiles like amines and nitriles (Scheme 2b,c), unmasking alcohol functionalities suitable for further reactions with acid chlorides, anhydrides, sulfonyl chlorides, chloroformates, carbamoyl chlorides and isocyanates (Scheme 2d). Finally, reductive N-O bond cleavage provides two additional orthogonal sites for functionalization, although this opportunity was not exploited.

**Scheme 3:**
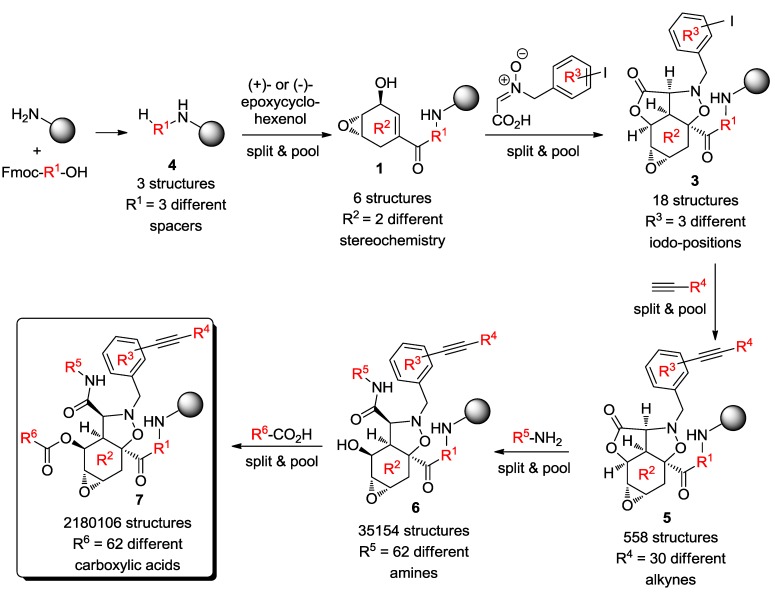
Split-and-pool DOS synthesis of the library based on template **3** [37].

Starting from these possibilities, the authors chose the most efficient reactions compatible with solid-phase split-and-pool technique as a synthetic strategy of choice to provide the greatest number of small molecules as possible [43,44]. According to this technique, the starting materials are linked to the solid support and divided into different batches (*split*); each batch is allowed reacting with the subsequent building blocks and the products are mixed again (*pool*). In this way, the number of products increases exponentially, affording thousands of structures, as required for the successful outcome in chemical genetics screening programs. Although this technique presented some difficulties in purification processes and in developing a validation protocol, Schreiber and co-workers were able to construct a DOS library of more than 2 million distinct, spatially separated, chemical entities [36,37]. As shown in Scheme 3, the resin was split into three portions and labelled with three different spacers (ω-aminocaproic acid, glycine and “no spacer”). The functionalized resin **4** was pooled, mixed and split in two equal portions. Each enantiomer of epoxycyclohexenol was coupled to each portion, thus resulting in six structures for **1**. The inclusion of *o*-, *m*-, *p*-iodobenzyl nitrone carboxylic acids led to 18 tetracyclic templates for **3**. Then, a Sonogashira/Castro-Stephens coupling reaction employing 30 different commercially available alkynes, afforded 558 compounds for **5**. Lactone aminolysis with 62 different amines resulted in 35,154 structures for **6**, and finally, alcohol acylation with 62 different carboxylic acids led to a DOS library of 2,180,106 distinct molecules of general formula **7**.

The solid-phase technique can be arranged efficiently with cytoblot assays [45] and small molecule microarray systems [46,47] for forward and reverse chemical genetics studies, respectively. In order to take full advantage of this feature, assay formats must be developed using a controlled release from the individual supports into distinct plate wells. For example, the authors developed instrumentations and robotics to array efficiently synthesis beads into distinct wells containing mink lung cells. With this miniaturized cytoblot assay, they found that all the library compounds showed a significant inhibitory effect on cell proliferation [37]. Additionally, in a preliminary study, they discovered that several compounds activate a TGF-β (Transforming growth factor β) responsive reporter gene in mammalian cells [48]. TGF-β induces cell cycle arrest in G1, so new compounds targeting TGF-β signaling components may be considered as hit compounds for the discovery of anti-cancer or anti-angiogenic agents.

**Scheme 4:**
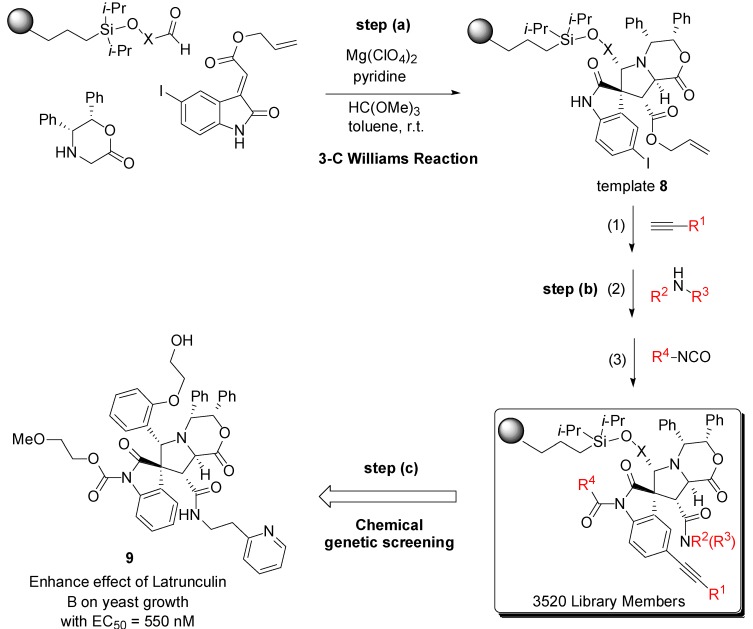
(**a**) Stereoselective synthesis of the spirooxindole scaffold **8**; (**b**) Scaffold decoration by cross-coupling reactions (1), amide formations (2), *N*-acylations (3); (**c**) From this library, a potent enhancer of latrunculin B was discovered [49].

In a further study, Schreiber and co-workers repeated the same synthetic strategy [49]. They reported the split-and-pool synthesis of more than 3000 spirooxindol compounds, exploiting a three-component Williams’ coupling reaction [50,51] to assemble the central core **8** (Scheme 4, step *a*).

Scaffold **8**, similarly to the previous template **3**, is a densely functionalized structure that allows for the application of several approaches for appendage’s decorations. The authors obtained a library of 3520 members using palladium-catalyzed coupling reactions (1), amide bond formations on the unmasked alcohol (2), and *N*-acylations of the γ-lactam (3) (Scheme 4b). A cell-based screening was then performed on this compounds collection to identify enhancers of latrunculin B, an actin polymerization inhibitor, which induces yeast growth arrest [52]. Through follow-up synthesis, the hit compound **9** as in Scheme 4, (step c) was confirmed and found to be a latrunculin B enhancer with an EC_50_ value in the sub-micromolar range.

## 3. Case Study 2: Contribution of David R. Spring to the Discovery of Antimitotic Agents through Chemical Genetics Screening of DOS Libraries

**Scheme 5:**
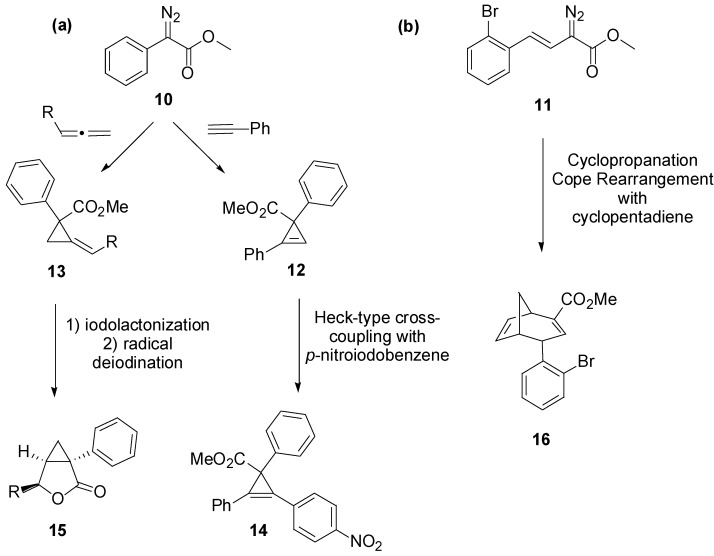
Scaffold generated from phenyldiazoester compounds **10** (**a**) and **11** (**b**) [53].

**Scheme 6:**
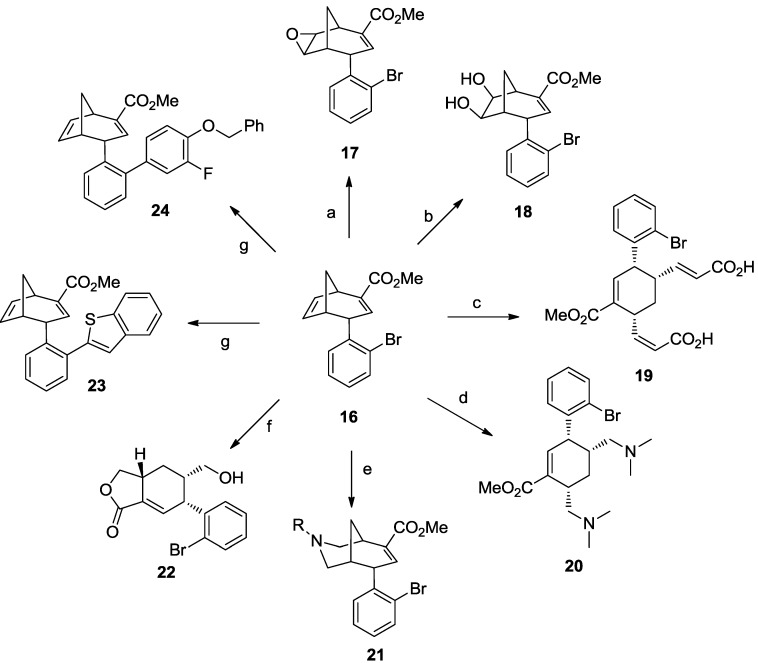
Library synthesis from the key intermediate **16**. The exploration of the chemical space around scaffold **16** was achieved using (**a**) epoxidation, (**b**) dihydroxylation, (**c**) ring opening metathesis, (**d**) dihydroxylation/oxidative cleavage + reductive amination with dimethylamine, (**e**) dihydroxylation/oxidative cleavage + double reductive amination with primary amines, (**f**) dihydroxylation/oxidative cleavage + esterification, (**g**) Suzuki reactions [53].

Recently, the group of David Spring reported a striking diversity-oriented synthesis combined to a forward chemical genetics study [53]. In this work, they obtained a DOS library of 35 compounds based on 10 distinct molecular scaffolds exploiting rhodium carbenoid chemistry. As shown in Scheme 5a, phenyldiazoester **10** reacts with terminal alkynes and alkenes providing cyclopropene (**12**) and cyclopropane structures (**13**), respectively, which can be further functionalized into scaffold **14** and scaffold **15**. The styril diazoester derivative **11** (Scheme 5b) revealed to be more interesting to access diversity in the DOS library through a tandem cyclopropanation-Cope rearrangement reaction [54], thus providing the multivalent bicyclo[3.2.1]octadiene **16**. In fact, the presence of both an electron-deficient and an electron-neutral double bond moiety, together with the proximal aryl bromide and a carboxylic acid ester, gave the opportunity to modify regioselectively the scaffold in a multidirectional divergent approach (Scheme 6).

For example, compound **16** was converted to epoxide **17** by a stereoselective olefin epoxidation (Scheme 6a), whereas stereoselective dihydroxylation of the electron-neutral double bond led to *cis*-diol **18**, which can be further transformed to acetals or sulfoxides (Scheme 6b). Additionally, the electron-neutral alkene moiety in **16** was exploited for ring opening metathesis with terminal olefins, affording cyclohexene structures such as compound **19** (Scheme 6c). The application of **16** to one-pot dihydroxylation/oxidative cleavage methodology [55], followed by reductive amination conditions of the dialdehyde intermediate, resulted to a new ring system. In particular, when **16** was left reacting with dimethylamine (Scheme 6d), the cyclohexene scaffold **20** was synthesized, whereas the use of primary amines resulted in a 6-6 fused ring system (compound **21**) as a consequence of double reductive amination (Scheme 6e). Additionally, the dialdehyde intermediate was selectively reduced to diol and cyclized under transesterification reaction conditions, thus obtaining the lactone **22** in just one synthetic step (Scheme 6f). Finally, the ortho-bromoaryl substituent of **16** provided a functional handle for Suzuki cross-coupling reactions (Scheme 6g), introducing aryl substituents around the core scaffold (compounds **23** and **24**).

A very useful tool for describing quantitatively the molecular diversity of DOS library compounds in the chemical space is the application of chemoinformatics [56,57,58]. Chemical space, as described by Dobson, is “the total descriptor space that encompasses all the small carbon-based molecules that could in principle be created” [59]. To computationally assess the structural diversity of a library and its ability to interrogate the chemical space, two comparative statistical approaches are typically employed: the principal component analysis (PCA) [60,61,62] and the principal moment of inertia (PMI) analysis [63,64].

**Figure 4:**
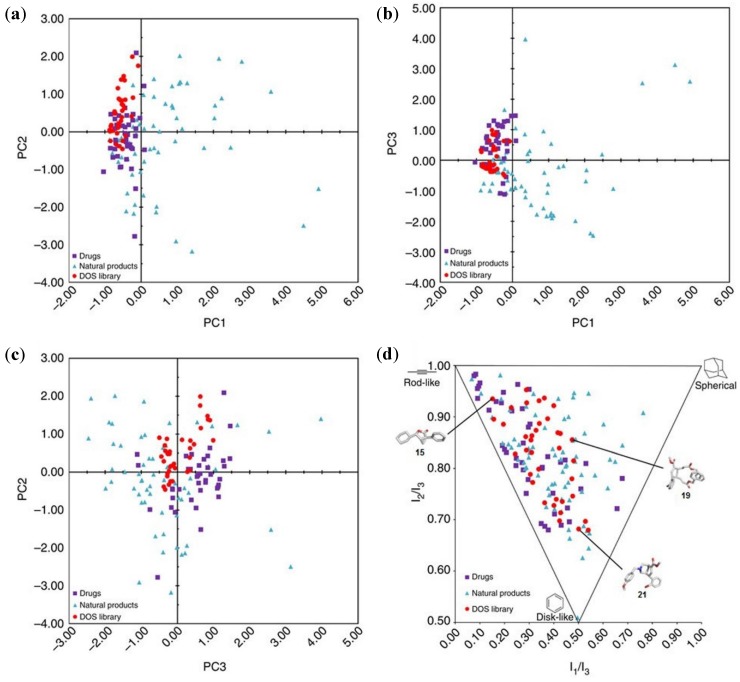
(**a**–**c**) Comparative PCA plots of DOS library compounds (red, circles) *vs*. top-selling brand-name drugs (purple, squares) and natural products (blue, triangles); (**d**) PMI plot illustrating the molecular shape diversity of the DOS library (red, circles) and lowest-energy conformation of three representative DOS library members (**15**, **19**, **21**). Adapted by permission from Macmillan Publishers Ltd: ref. 53, copyright (2014).

PCA utilizes a defined number of descriptors (such as molecular weight, logP values, number of H-bond donors or acceptors) to represent each molecule as two-dimensional vectors, which can be plotted to give a graphic representation of the library diversity. Spring and co-workers analysed 15 physicochemical properties of the compounds by comparing them with two reference compound collections, a pool of 40 top-selling brand-name drugs and a pool of 60 bioactive natural products [62]. PCA graphs (Figure 4a–c) showed that the DOS library overlaps considerably with the chemical space covered by the top-selling drugs, indicating the potential drug-likeness of these scaffolds.

Normalized ratios of principal moments of inertia (PMI) represent an intuitive way to describe molecular shape. The authors plotted the PMI normalized ratio of the minimum energy conformation of each library member into a triangular graph. The obtained PMI graph, with its vertices representing the three extremes of molecular 3D geometry (rod, disc, sphere), showed that the DOS library has a very good level of shape diversity (Figure 4d). Additionally, DOS compounds tend towards the spherical corner more than drugs, suggesting that their scaffolds are more three-dimensional in character than drugs. This is a very interesting feature, because an increase in the three-dimensional and stereochemical complexity enhances the chance of modulating challenging targets, such as protein-protein and protein-DNA interactions [65].

**Figure 5:**
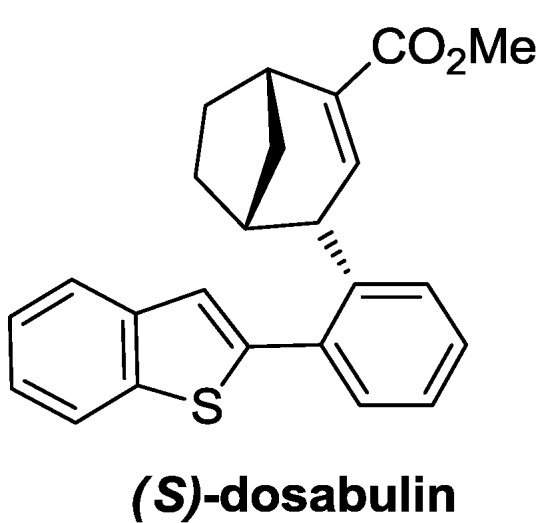
The antimitotic compound, (*S*)-Dosabulin, discovered with forward chemical genetics approach [53].

The DOS library was then assayed in a microscopy-based phenotypic screening [66] for mitotic arrest, thus finding that compounds **23** and **24** induce a marked cell arrest in the mitosis. Starting from these results, the authors synthesized a partially saturated analogue of compound **23**, named dosabulin (Figure 5), discovering that all the activity was located in the (*S*)-enantiomer. Furthermore, according to a forward chemical genetics approach, the authors tried to identify the target responsible for the mitotic arrest induced by (*S*)-dosabulin. Since the tubulin network was demolished by treatment with (*S*)-dosabulin, they suggested that dosabulin targets tubulin itself, as several antimitotic drugs do [67].

This work revealed an additional advantage of the forward chemical genetics approach. In fact, it is often possible to identify compounds that perturb other cell functions, as compared to the phenotype of study, and in this case the authors identified two other compounds, based on the scaffold **21** (Scheme 6), able to decrease cell viability, without inducing a mitotic arrest. Their identification is a further demonstration of the ability of DOS to produce useful bioactive scaffolds with potential therapeutic applications.

## 4. Case Study 3: Our Contribution to the Chemical Genetics Screening of Peptidomimetic DOS Libraries towards Yeast Deletant Strains

From the current literature, it’s worth noting that the rate-limiting step of DOS-chemical genetics studies is the development of an easy and economical screening process rather than the design of a DOS library [68]. In this context, our group, in collaboration with the research group of Duccio Cavalieri, found that a useful tool for forward chemical genetics programs is the use of *Saccharomyces cerevisiae* [69]. Thanks to its easy manipulation and its rapid life cycle, this model organism can be successfully used to identify novel compounds inducing cell growth inhibition [70]. Furthermore, in addition to the high degree of conservation with mammalian cells, *S. cerevisiae* is particularly suited for dissecting biological pathways and for identifying novel targets. Once active compounds have been selected, the screen of yeast mutant strains, showing hypersensitivity or hyper-resistance to each molecule, allows for the identification of the target responsible for the observed phenotype [71,72,73,74]. Specifically, the deletant strain that is not perturbed by the presence of such a compound is the one that lacks the gene encoding for the target of the molecule.

Using this approach, together with Cavalieri’s group, we developed forward chemical genetics methods using DOS libraries of morpholine-based peptidomimetics [75,76,77]. A peptidomimetic compound may be defined as a molecule having a secondary structure similar to the parent peptide, such that it binds to enzymes or receptors with higher affinity than the starting peptide. These compounds, able to perturb protein-protein interactions, have attracted wide interest in chemical genetics studies for discovering protein function and identifying novel ligands [78,79]. In this context, our group reported several efficient synthetic strategies for the production of peptidomimetic templates characterized by polyfunctional and heterocyclic structures, starting from amino acid and sugar derivatives [80,81,82,83].

### 4.1. Chemical Genetics of a Bicyclic Peptidomimetic DOS Library

**Figure 6:**
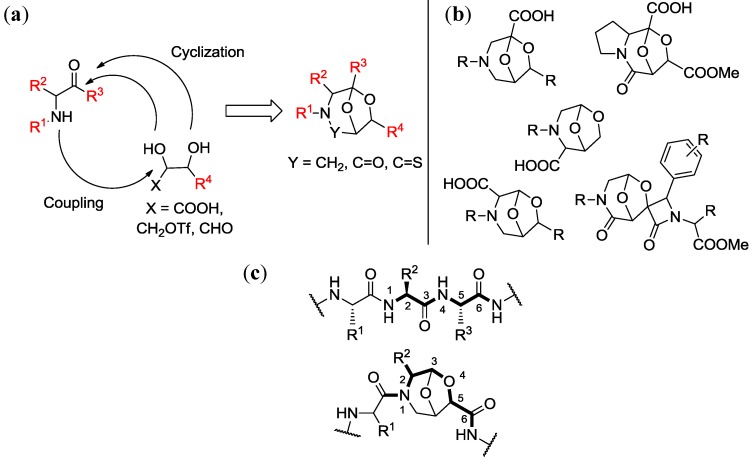
(**a**) Strategic approaches for the synthesis of peptidomimetic scaffolds starting from sugar and amino acids derivatives; (**b**) Representative examples of bicyclic rigid scaffolds obtained with this strategy; (**c**) 6,8-dioxa-3-azabicyclo[3.2.1]octane scaffold as a constrained dipeptide isostere [80,81,82,83].

In a first work [75], we developed a chemical genetics study based on a library of bicyclic peptidomimetics characterized by a 6,8-dioxa-3-azabicyclo[3.2.1]octane core. This collection of compounds was generated, as shown in Figure 6a, through two key steps consisting of a coupling reaction between two building blocks followed by an intramolecular cyclization [80,81]. By tuning the starting materials it was possible to generate a large array of different scaffolds (Figure 6b). These templates show structural similarity to dipeptides through an atom-by-atom correlation, in fact the side chain can be placed in the same position as found in native dipeptide sequences (Figure 6c). Additionally these compounds satisfy all the requirements needed for the peptidomimetic chemistry, as they are easily synthesized and decorated with functional groups, and are well-suited for solid-phase synthesis. These scaffolds revealed to be active hit compounds towards different targets, as they have been used as aspartyl protease inhibitors [84,85] and NGF-agonists [86].

**Figure 7:**
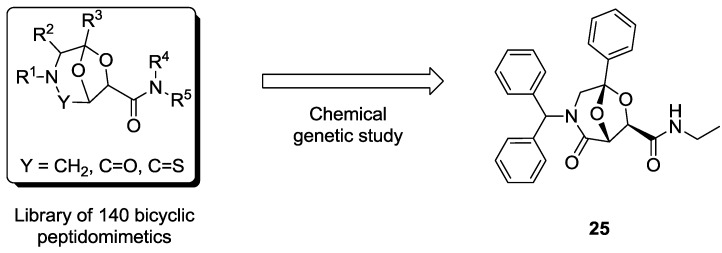
From a library 140 bicyclic peptidomimetics, a forward chemical genetics study allowed for the identification of **25** as a hit antifungal compound [75].

In a preliminary chemical genetics study, a library composed of 140 bicyclic peptidomimetics (Figure 7) was tested on a panel of *S. cerevisiae* wild-type strains, using cell growth as the phenotype of study. Sixteen compounds resulted in a decrease of more than 10% of the growth rate. In order to gain insight into their mode of action, these selected compounds were re-tested on a panel of mutant strains, harbouring a *HAP1* gene deletion that resulted in limited respiratory ability [87]. This process enabled the selection of compound **25** (Figure 7), which was identified as the molecule inducing the most intense cell growth decrease. This peptidomimetic was more active on the wild-type strain than on the *HAP1*-deleted one, thus giving an indication of the involvement of the respiratory metabolism in response to perturbation operated by compound **25**.

The pathway signature-based approach [88,89], which compares the transcriptional profile of wild-type and mutant cells, has proven to be a very useful method to validate drug targets and to identify secondary effects. According to this approach, the pathway analysis of gene expression levels of mutant and wild-type yeasts confirmed *HAP1* as one of the targets of compound **25**.

Furthermore, the use of pool of strains bearing deletion of cell wall and multidrug resistance genes (*ERG6*, *SNQ2* and *PDR3*) indicated that **25** induces other biological effects, such as the generation of an important stress condition. Additionally, these data enabled the localization of the first molecular target of compound **25** at the cell wall level. Since cell wall is a feature of yeast, compound **25** can be considered a potential selective antifungal agent.

### 4.2. Chemical Genetics of Morpholine-Based DOS Library

**Scheme 7:**
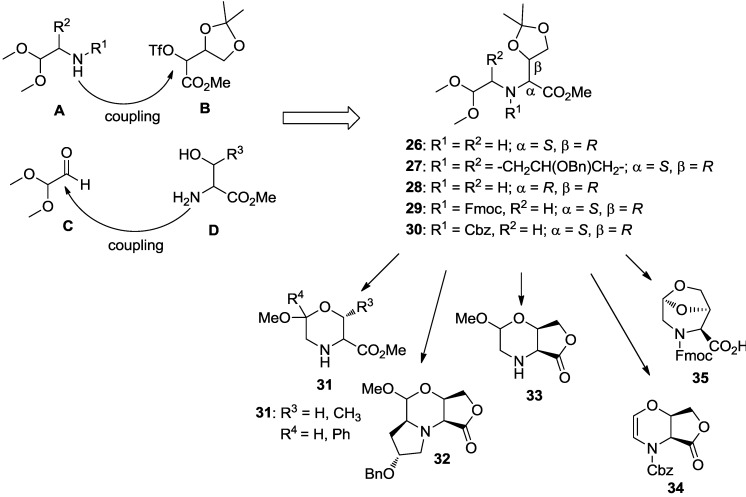
Selected building blocks for the coupling step and skeletal diversity resulting from the cyclization step [90].

In a further chemical genetics work [76], we generated a DOS library of morpholine-based compounds to be screened towards yeast strains. According to the build/could/pair approach, we developed a DOS strategy for the generation of morpholine scaffolds, taking advantage of a two-steps process. The strategic coupling of the four derivatives **A**–**D** as shown in Scheme 7, resulted to a highly functionalized acyclic precursor.

This intermediate revealed to be very useful for accessing diversity around the morpholine nucleus. In fact, the selection of building blocks and the choice of cyclization conditions, allowed for obtaining several skeletally different scaffolds [90]. For example, compounds **26** and **27** gave bicylic lactones **31** and **32**, respectively, upon treatment with a methanolic SOCl_2_ solution. Instead, compound **28**, as a consequence of steric bias, could not cyclize under the same reaction conditions, and gave the polyfunctional morpholine scaffold **33**. Furthermore, starting from compounds **29** and **30**, containing the protected amino group, oxazine **34** and the bicylic scaffold **35** were obtained (Scheme 7).

**Scheme 8:**
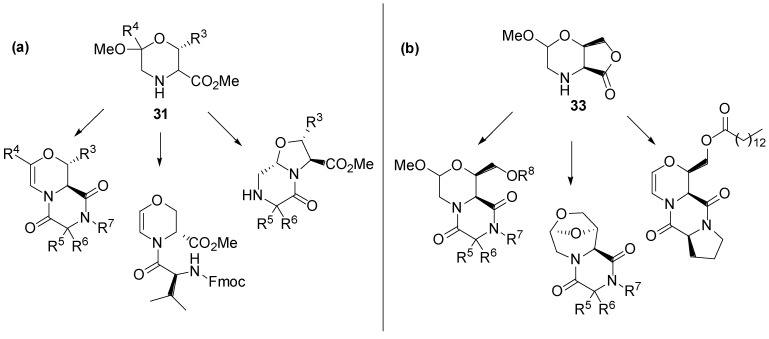
Representative examples of library members obtained (**a**) from scaffold **31** and (**b**) from scaffold **33** [76].

From scaffold **31** and **33**, a library of 48 distinct compounds, embedding 2,5-diketopiperazine, 2-oxopiperazine and 1,4-dihydrooxazine heterocycles, was generated (Scheme 8). Such skeletal diversity was accessed exploiting the reactivity of morpholine acetals as a function of the reaction methodology [91]. The compounds of this library contain interesting biochemical features, such as the morpholine moiety, which is present in several bioactive molecules [92,93], and the 2,5-diketopiperazine nucleus, which is considered a privileged scaffold in medicinal chemistry [94,95].

**Figure 8:**
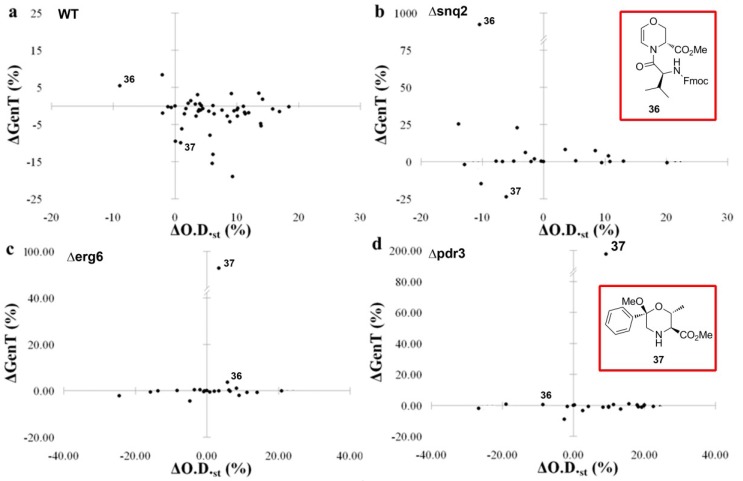
Bidimensional scatter plots showing effects of library members on cell growth of selected deletant strains and structures of compounds **36** and **37** [76]. Cell growth inhibition on (**a**) wild-type strain; (**b**) ∆*snq2* deletant strain; (**c**) ∆*erg6* deletant strain; (**d**) ∆*pdr3* deletant strain.

The effects of these 48 library members were tested on *S. Cerevisiae* wild-type strain. In order to improve the profile of phenotype screening, yeast cell growth was evaluated in both exponential and stationary phases. This screening allowed for the selection of 21 molecules inducing a variation in the O.D._650_ value, which is correlated to the number of cells in the stationary phase, and/or in GenT, which is the time responsible for cell division in the initial phase. Such compounds were thus tested on both wild-type (WT) and mutant strains bearing deficiencies in genes involved in cell wall remodelling (∆*erg6*) and multidrug resistance (∆*snq2* and ∆*pdr3*), to gain insight into their mode of action, as described above (Figure 8).

The bidimensional scatter plots, as showed in Figure 8, allowed to rapidly identify eight compounds displaying the most prominent activity. Specifically, while compound **36** showed modest inhibition on both phases of cell growth (upper-left quadrant of Figure 8a), and compound **37** gave modest activation of cell growth and no inhibition of the stationary phase (Figure 8a), the activity on mutant strains resulted in dramatic inhibitory effects. Compound **36** proved to reduce strongly (about tenfold) the initial phase of cell growth (Figure 8b), which is related to cell replication in the ∆*snq2* deletant strain having defects in the extrusion pathways, as Snq2p is a drug-efflux pump ABC (ATP-Binding Cassette) transporter conferring resistance to drugs and oxygen radicals. Compound **37** showed 100% and 200% reduction of cell growth in the initial phase (see vertical axis of Figure 8c,d) in both ∆*erg6* and ∆*pdr3* deletant strains, bearing deficiencies in genes involved in cell wall and MDR (Multi Drug Resistance), respectively. Such effects gave initial clues on the role of compound **37** in targeting the ergosterol biosynthesis pathway downstream Erg6p.

Following the screening of eight selected compounds, including compounds **36** and **37**, for the mitochondrial membrane potential activation and peroxisomal proliferation, the two hit compounds **36** and **37** were successively validated. Compound **36**, may act as a starvation inductor causing respiration and β-oxidation decrease. On the other hand, compound **37**, inducing the lowest peroxisomal proliferation is a promising molecular probe to dissect the drug resistance mechanism in yeast and mammalian cells.

## 5. Conclusions

The application of chemical genetics in drug discovery research is becoming increasingly popular, as it holds the promise of discovering both new targets and new lead compounds. In order to make this kind of study valid, a suitable phenotype screening and versatile synthetic methods are needed. Diversity-oriented synthesis, as shown by the herein presented case studies, contributed significantly to the development of large small molecule collections. Nevertheless, greater efficiency is still required, especially for purifying and arraying library compounds in an arrangement suitable for screening processes. This interdisciplinary profile is striking, as it consists of harmonizing synthetic methodology with new analytical and biological methods. On the other hand, the whole screening apparatus does not satisfy yet the chemical genetics requirements. Considerable efforts are needed for developing new deconvolution methods, in order to decipher the mode of action of active compounds and to identify novel molecular targets. If the difficulties regarding the screenings will be solved, chemical genetics will expand significantly our knowledge of the druggable genome, opening the way to better chemotherapeutic treatments.
